# Supplementary material for: Effectiveness of two extended-release buprenorphine formulations during postoperative period in neonatal rats
Source: PLoS One. 2022 Oct 17;17(10):e0276327. doi: 10.1371/journal.pone.0276327 (PMC9576048; doi:10.1371/journal.pone.0276327)
Supplement: Attachment — Submitted filename: Dear author.docx [file pone.0276327.s002.docx]

*Dear author.*

*If the authors consider the following points, the manuscript will become more valuable:*

1. *The title is inconsistent; if possible, rewrite it to make it moreclear.*
2. *The abstract is vague and inconsistent; the goal, experiment protocol, and groups are unclear; it should be rewritten. Abbreviations such as XR-Hi, XR-Lo, and XR should be placed appropriately. Introduction: from lines 50-53, these are results, that must be put in the result section.*

*Add some lines for the differences between Bup-SR and Bup-XR and why the author chooses these doses.*

3. *Experimental groups: lines 84-85 the dose of the group is 0.65 mg/ kg low dose extended-release buprenorphine (XR-Lo; ), but write 1.3 mg/kg, please confirm which dose is correct. Additionally, this section needs rewriting to be consistent. Why does the author use these doses?*
4. *Anesthesia and surgical section: lines 93-95, Then all experimental treatments and supplemental fluid (0.9% NaCl, 5 ml/kg) were administered SC at the left and right shoulders, respectively. This section is not obvious, please rewrite this section.*
5. *Thermal Hypersensitivity Testing: in line 116, write 700 AM, what are the authors mean here? Furthermore, this section is vague.*
6. *In plasma collection: this part of the study why separated from the first part?*
7. *Plasma Concentration Analysis: abbreviation of DI to what refer?*
8. *Results: In line 214, the authors write there is a significant decrease in the thermal hypersensitivity in times 1,4, and 8 hrs, but in figure 2 A there is an increase in the thermal hypersensitivity, please give an explanation for this increment. Please, all results need checking.*
9. *Discussion:*
a. *The aim written in line 264 differs from that in the introduction and abstract. Confirm which one is correct.*
b. *Write whole word before abbreviation of Ethiqa-xr.*
c. *Many sentences are repeated frequently in the manuscript, so, revisions are required. See lines 273- 275 and the paragraph before these lines as an example.*
d. *The authors write the tested drug is safely and effectively attenuated the thermal hypersensitivity, please how measure these parameters?*
e. *Lines 285-287 are related to the materials and method section.*
f. *The word Saline should be used in uniform, because some times write with capital letter and other times with small letter.*
g. *From line 296 to 310 are related to the introduction section.*
h. *Lines 325- 337 are repeated and related to results section.*

*With best regards,*
