# Supplementary material for: Effectiveness of two extended-release buprenorphine formulations during postoperative period in neonatal rats
Source: PLoS One. 2022 Oct 17;17(10):e0276327. doi: 10.1371/journal.pone.0276327 (PMC9576048; doi:10.1371/journal.pone.0276327)
Supplement: Attachment — Submitted filename: Zhang et al. rebuttal letter.docx [file pone.0276327.s004.docx]

September 27, 2022

Dr. Sairah Hafeez Kamran

Academic Editor

PLOS ONE

Re: Manuscript ID PONE-D-22-06559R1

Dear Dr. Sairah Hafeez Kamran,

Thank you for your letter of September 26, along with the comments of the reviewers. We thank the reviewers for their constructive feedback on our manuscript.

Our detailed point-by-point responses to reviewer concerns are presented below and we have revised the manuscript accordingly.

Please let me know if further clarification is required. I can be reached by phone (650) 497-1905, or via email mynzhang@stanford.edu.

Sincerely,

Mingyun Zhang

ACADEMIC EDITOR:

I will suggest to increase the clarity of discussion by incorporating scientific explanations of the research question and correct minor grammatical mistakes. The resolution of figures shall be increased to enhance clarity.

**Response**: We revised our Discussion section accordingly. The manuscript was proofread, and grammatical mistakes are corrected. The resolution of figures has been increased.

Reviewers' comments:

Reviewer's Responses to Questions

**Comments to the Author**

1. If the authors have adequately addressed your comments raised in a previous round of review and you feel that this manuscript is now acceptable for publication, you may indicate that here to bypass the “Comments to the Author” section, enter your conflict of interest statement in the “Confidential to Editor” section, and submit your "Accept" recommendation.

Reviewer #1: (No Response)

Reviewer #2: All comments have been addressed

2. Is the manuscript technically sound, and do the data support the conclusions?
The manuscript must describe a technically sound piece of scientific research with data that supports the conclusions. Experiments must have been conducted rigorously, with appropriate controls, replication, and sample sizes. The conclusions must be drawn appropriately based on the data presented.

Reviewer #1: Partly

Reviewer #2: Yes

**Response**: We have revised accordingly.

3. Has the statistical analysis been performed appropriately and rigorously?

Reviewer #1: I Don't Know

Reviewer #2: Yes

**Response**: Our statistical analysis was performed by Dr. Katechan Jampachaisri, a statistician, from the Department of Mathematics, Naresuan University.

4. Have the authors made all data underlying the findings in their manuscript fully available?
The [PLOS Data policy](http://www.plosone.org/static/policies.action#sharing) requires authors to make all data underlying the findings described in their manuscript fully available without restriction, with rare exception (please refer to the Data Availability Statement in the manuscript PDF file). The data should be provided as part of the manuscript or its supporting information, or deposited to a public repository. For example, in addition to summary statistics, the data points behind means, medians and variance measures should be available. If there are restrictions on publicly sharing data—e.g. participant privacy or use of data from a third party—those must be specified.

Reviewer #1: Yes

Reviewer #2: Yes

5. Is the manuscript presented in an intelligible fashion and written in standard English?
PLOS ONE does not copyedit accepted manuscripts, so the language in submitted articles must be clear, correct, and unambiguous. Any typographical or grammatical errors should be corrected at revision, so please note any specific errors here.

Reviewer #1: No

Reviewer #2: Yes

**Response**: We revised the manuscript and typographical or grammatical errors were corrected.

6. Review Comments to the Author

Please use the space provided to explain your answers to the questions above. You may also include additional comments for the author, including concerns about dual publication, research ethics, or publication ethics. (Please upload your review as an attachment if it exceeds 20,000 characters)

Reviewer #1: Dear author,
Many sections of the manuscript are still vague and uninformative. The abstract is still vague and lacks a conclusion.
with regards,

**Response**: We revised the Abstract and Discussion sections.

Reviewer #2: Authors have made the required changes in the manuscript as suggested. Abstract is looking better now. I am satisfied with the explanation of authors regarding novelty of the research question. I will suggest to include this explanation in discussion to reveal its novelty and mentioning the limitations. I will also suggest to improve the resolution of the figures.

**Response**: We revised Discussion section accordingly. We also improved the resolution of the figures.
